# Therapeutic Targeting of Glutaminolysis as a Novel Strategy to Combat Cancer Stem Cells

**DOI:** 10.3390/ijms232315296

**Published:** 2022-12-04

**Authors:** Ting-Wan Kao, Yao-Chen Chuang, Hsin-Lun Lee, Chia-Chun Kuo, Yao-An Shen

**Affiliations:** 1 Department of Pathology, School of Medicine, College of Medicine, Taipei Medical University, Taipei 110301, Taiwan; 2 Graduate Institute of Clinical Medicine, College of Medicine, Taipei Medical University, Taipei 110301, Taiwan; 3 Department of Radiation Oncology, Taipei Medical University Hospital, Taipei 110301, Taiwan; 4 Department of Radiology, School of Medicine, College of Medicine, Taipei Medical University, Taipei 110301, Taiwan; 5 Taipei Cancer Center, Taipei Medical University, Taipei 110301, Taiwan; 6 School of Health Care Administration, College of Management, Taipei Medical University, Taipei 110301, Taiwan; 7 Ph.D. Program for Cancer Molecular Biology and Drug Discovery, College of Medical Science and Technology, Taipei Medical University and Academia Sinica, Taipei 11031, Taiwan; 8 International Master/Ph.D. Program in Medicine, College of Medicine, Taipei Medical University, Taipei 110301, Taiwan

**Keywords:** cancer stem cells, glutaminolysis, glutaminase, metabolic compensation, therapeutic resistance

## Abstract

Rare subpopulations of cancer stem cells (CSCs) have the ability to self-renew and are the primary driving force behind cancer metastatic dissemination and the preeminent hurdle to cancer treatment. As opposed to differentiated, non-malignant tumor offspring, CSCs have sophisticated metabolic patterns that, depending on the kind of cancer, rely mostly on the oxidation of major fuel substrates such as glucose, glutamine, and fatty acids for survival. Glutaminolysis is a series of metabolic reactions that convert glutamine to glutamate and, eventually, α-ketoglutarate, an intermediate in the tricarboxylic acid (TCA) cycle that provides biosynthetic building blocks. These building blocks are mostly utilized in the synthesis of macromolecules and antioxidants for redox homeostasis. A recent study revealed the cellular and molecular interconnections between glutamine and cancer stemness in the cell. Researchers have increasingly focused on glutamine catabolism in their attempt to discover an effective therapy for cancer stem cells. Targeting catalytic enzymes in glutaminolysis, such as glutaminase (GLS), is achievable with small molecule inhibitors, some of which are in early-phase clinical trials and have promising safety profiles. This review summarizes the current findings in glutaminolysis of CSCs and focuses on novel cancer therapies that target glutaminolysis in CSCs.

## 1. Introduction

According to estimates from the World Health Organization (WHO) in 2022, cancer will be the first or second leading cause of death worldwide, accounting for more than 18 million cases of cancer diagnosed and nearly 10 million deaths in 2020 [1]. Furthermore, the global cancer burden is expected to be 28.4 million new cancer diagnoses in 2040, a 47% rise from 2020 [2]. The mortality rate has steadily decreased since the 1990s, thanks to the rapid development of oncology research, improved understanding of pathologies, and the engineers and physicists who continuously impact the diagnosis and treatment technologies. Regular health checkups and a variety of treatment options, including surgery, chemotherapy, radiation therapy, targeted therapy, immunotherapy, stem cell or bone marrow transplant, and hormone therapy, can pave the way for an improved cancer prognosis and treatment alternatives. However, these standard strategies fail to eradicate cancer cells because they only kill differentiated cancer cells while sparing cancer stem cells (CSCs), which results in a burden of cancer incidence and mortality that rapidly grows worldwide.

CSCs are a subpopulation of cancer cells that have self-renewal and differentiation properties and have been shown to initiate cancer development, recurrence, and metastasis, as well as playing important roles in radio-, chemo-, and immunotherapy resistance [3,4,5,6,7,8,9,10]. The majority of tumor cells generate the majority of their energy through glycolysis, even in oxygen-rich environments, which is known as the Warburg effect [11]. However, CSCs have a unique metabolic flexibility that allows them to switch between glycolysis and oxidative phosphorylation (OXPHOS) to maintain homeostasis and meet high energy demands for tumor proliferation under adverse conditions such as hypoxia, acidosis, and starvation. In addition, they have unique metabolic demands relying on the utilization of specific amino acids and other nutrients, especially glutamine. Glutamine, the most abundant amino acid in plasma, functions as a key substrate in a number of different biosynthetic pathways, including the production of nucleotides (purines and pyrimidines), fatty acids, antioxidants, and other non-essential amino acids (NEAAs). Many of these processes are necessary for CSCs to function properly [12]. On the other hand, targeting glutaminolysis pathways, which include the tricarboxylic acid (TCA) cycle, the electron transport chain, and the pentose phosphate pathway, can be a promising approach to dampen radio- or chemo-resistant properties of CSCs and, therefore, increase the curability of tumors [13,14,15,16]. There has been a significant emphasis placed in cancer research on glutaminolysis-based therapy, and a significant amount of effort has been poured forth in the exploration of novel therapeutic targets.

In this article, we emphasize the essential players in the glutaminolysis pathways that are linked to CSCs, with a special focus on the glutaminolysis-based therapeutic techniques that have been deployed in preclinical and clinical contexts. Specifically, we focus on the glutaminolysis-based therapeutic strategies that have been utilized in clinical settings. Specific glutaminolysis inhibitors have the potential to override stem cell characteristics, which might minimize the risk of disease recurrence. These inhibitors could also discourage the spread of CSCs and the propensity of cancer cells to give rise to metastases.

## 2. Catabolism of Glutamine

**Figure 1:**
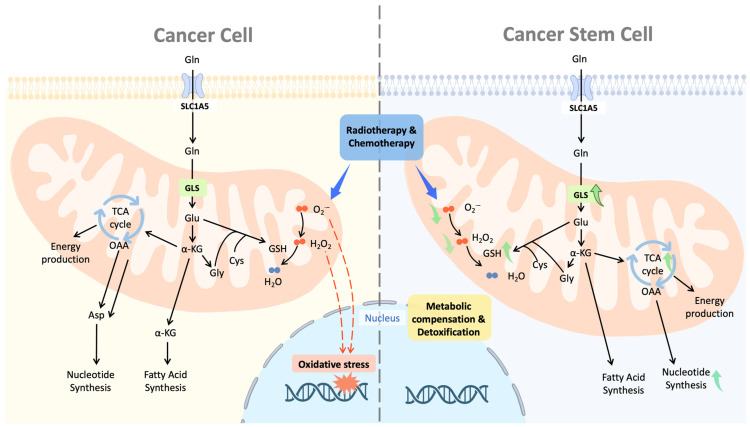
The metabolic compensation of CSCs. CSCs exhibit enhanced glutaminolysis as a result of the overexpression of glutaminase and the consumption of glutamine. This trait contributes to the metabolic compensation for chemotherapy by promoting the production of glutathione to scavenge free radicals and nucleotide biosynthesis to repair DNA damage. Both of these processes are necessary to compensate for the damage caused by chemotherapy. Additionally, glutathione has the ability to neutralize platinum medicines, thus lowering the drug concentration within cells. As a result of the metabolic compensation, CSC acquires the ability to resist therapy with conventional treatments.

Glutamine is a plentiful nutrient that plays a vital role in the generation of energy, the maintenance of redox balance, the synthesis of biological macromolecules, and the transmission of signals between cells [17]. The term “glutamine addiction” is widely used to reflect the strong glutamine dependence of most cancer cells for this essential nitrogen substrate after metabolic reprogramming. In CSCs, glutamine provides the source of carbon and amino nitrogen, which play an important role in manufacturing a wide variety of cellular components, including nucleic acids, lipids, amino acids, and the antioxidant glutathione (GSH) [18]. Through a redox-mediated mechanism and nucleotide biosynthesis to repair DNA damage, glutamine plays a crucial role in preserving the stemness of cancer cells (Figure 1). Both of these mechanisms are required to compensate for the damage induced by chemotherapy [19] (Figure 1). Furthermore, glutathione has the capability to neutralize platinum drugs, thereby reducing their concentration inside cells [20] (Figure 1). Resistance to therapy with conventional treatments develops in CSC as a result of metabolic compensation (Figure 1).

**Figure 2:**
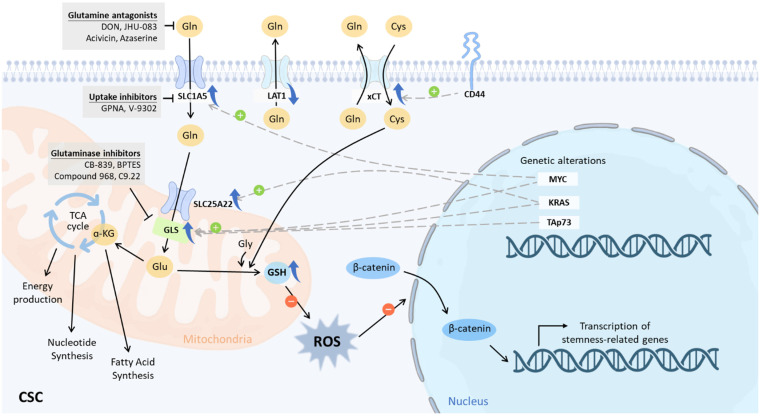
The glutaminolysis pathway and its inhibitors. Glutamine is the most prevalent amino acid in the blood and serves as a fuel source for the biosynthesis of fatty acids, nucleotides, and antioxidants in CSCs. MYC has the ability to raise the expression levels of the glutamine transporter ASCT2 as well as glutaminase. The glutaminolysis inhibitors include glutamine analogues such as DON, JHU-083, and acivicin azaserine, as well as glutaminase inhibitors such as CB-839, BPTES, compound 968, and C9.22. These inhibitors have the ability to prevent CSCs from engaging in metabolic compensation and from exhibiting aggressive phenotypes.

Glutaminolysis is a series of reactions in which glutamine is catabolized to produce substrates for macromolecular synthesis and energy. Glutaminolysis starts with the intake of glutamine into the cell via transporters such as alanine, serine, and cysteine transporter 2 (ASCT2, also known as SLC1A5), and glutamine can subsequently be converted to glutamate by glutaminase 1 (GLS1) or glutaminase 2 (GLS2) (Figure 2). Glutamate is then deamidated by glutamate dehydrogenase (GLUD) or several transaminases, yielding α-ketoglutarate (α-KG) for the TCA cycle [21]. Alanine and aspartate can also be produced by transaminase processes. In addition to being used in the energy-producing TCA cycle, α-KG can be exported from the mitochondria to the cytosol, where it is converted to isocitrate by isocitrate dehydrogenase 1 (IDH1) and then to citrate for de novo fatty acid synthesis [21,22] (Figure 2). Other compounds that facilitate cancer cell proliferation can be made from glutamine’s TCA cycle metabolites after they are released into the cytosol. Other TCA cycle metabolites produced from glutamine can also be transported to the cytosol and then be converted into other molecules. For example, the reduction capacity of cells can be refilled with malate by generating NADPH and pyruvate via malic enzyme 1 (ME1) in the cytosol (Figure 2). In nucleotide synthesis, oxaloacetate (OAA) and glutamate are transaminated to produce α-KG and aspartate [23,24,25] (Figure 2). There has been accumulating evidence to show that increased glutamine metabolism in cancer cells promotes tumorigenesis and cancer cell survival by maintaining redox homeostasis. GSH, an endogenous antioxidant that protects cancer cells from oxidative stress and enables them to survive, is produced from glutamine [12] (Figure 2). Glutamine contributes to redox hemostasis and provides nitrogen for nucleobase synthesis and the TCA cycle [26]. Glutaminolysis is on the rise as a target for the creation of novel small molecule anti-cancer medicines since it is quintessential for the survival and propagation of cancer cells [27,28].

A transcriptional pathway that is controlled by MYC is responsible for the stimulation of glutaminolysis. As a result, the mitochondrial TCA cycle becomes dependent on glutamine as a result of MYC’s activity [29]. MYC is also responsible for the activation of SLC38A5 (SN2), a major transporter of glutamine, as well as SLC1A5 (ASCT2), an amino acid transporter that is implicated in glutamine-dependent mammalian target of rapamycin complex 1 (mTORC1) activation. It has been demonstrated that the amounts of glutamine present in the cell have an effect on the protein translation regulator known as mTORC1 [30]. Through the simultaneous import of leucine into cells via the bidirectional transporter SLC7A5, glutamine is able to activate the mTORC1 signaling pathway [31]. Imported leucine binds to Sestrin2 and impairs the connection between Sestrin2 and GATOR2, consequently recruiting mTORC1 to lysosomes [31] (Figure 2). Glutamine can also activate mTORC1 via a Rag GTPase–independent pathway requiring ADP-ribosylation factor 1 (Arf1) [32] (Figure 2). The link between glutamine metabolism and mTORC1 activation also involves both an acute route that depends on glutamine-derived α-KG (Figure 2) and a secondary route that depends on ATP and AMPK [33]. Moreover, mTORC1 promotes glutamine anaplerosis via suppressing SIRT4 transcription to activate glutamate dehydrogenase (GDH) [34] (Figure 2). Additionally, augmented SLC1A5 is frequently found in cells that are reliant on glutamine [35]. For instance, SLC1A5 is abundantly expressed in triple-negative breast cancer (TNBC) patients, and tumor-bearing mouse survival was enhanced by knocking it down [36]. In addition to this, researchers have shown that the levels of glutaminase are elevated in cells that have an excessive amount of MYC expression [19,37]. Given that MYC, SLC1A5, and mTORC1 levels are higher in glutamine-addicted cancer cells, along with glutaminase levels, all of these can be exploited in future clinical studies as biomarkers to identify patients when assessing the potential effectiveness of glutaminolysis inhibitors.

## 3. Cancer Glutaminolysis

### 3.1. Alteration of Systemic Glutamine Utilization by Cancer

Glutamine is a versatile nutrient that is essential for the proliferation and survival of a large subset of cancer cells. To promote a better understanding of glutamine handling in cancer, enormous attention has been devoted to unraveling the inter-organ dynamics of glutamine metabolism. In normal physiology, the major source of plasma glutamine is skeletal muscle, while the major consumers of glutamine are the gastrointestinal tract and kidney. In times of stress, the lungs are also capable of releasing a marked amount of glutamine in response to corticosteroid-induced induction of glutamine synthase expression. While the liver has the capacity to metabolize glutamine, it is not a major contributor to the systemic glutamine pool under normal circumstances. Interestingly, cancer has a tendency to cause significant changes in the way glutamine circulates throughout organs in the body. Multiple studies have demonstrated that cancer has the capacity to markedly enhance the release of glutamine into circulation by skeletal muscle via upregulating glutamine synthetase. As tumor masses enlarge, the intramuscular glutamine reserve continues to be depleted, resulting in a loss of lean muscle mass and the development of a cachectic phenotype. In this scenario, the liver and kidneys would become net exporters of glutamine [17].

### 3.2. The Significance of Glutamine Metabolism for Cancer Survival

Reflecting the highly heterogeneous nature of cancer metabolism, the degree of glutamine dependence differs among cancers. However, it has been observed that certain cancer types may be more prone to glutamine addiction than others. These cancers entail pancreatic cancer, acute myelogenous leukemia, glioblastoma, myeloma, clear cell renal cell carcinoma, lung cancer, and TNBC [30,36,38,39,40].

Although glutamine is not an essential amino acid, it is necessary for the survival of cancer cells that are addicted to glutamine, and glutamine deficiency halts the growth of cancer [12]. Glutamine metabolism is known to be closely interlinked with the function of multiple oncogenic pathways and plays an essential role in the survival of cancer. For example, p53, a key tumor suppressor that is frequently mutated in human cancers, is known to boost the utilization of glutamine under conditions of low glucose and high reactive oxygen species (ROS), which maintains a redox equilibrium and ATP supply in cancer [41]. In response to glutamine deprivation, the arginine transporter SLC7A3 upregulates p53, and its expression encourages an increase in intracellular arginine levels, which increases the growth of tumors [42]. In order to shield cells from oxidative damage and genomic instability, p53 also stimulates the production of GLS2, which ultimately aids in the removal of intracellular ROS [43]. In the meantime, the lack of glutamine led to a reduction in tumor development in cancer cells that do not express p53 [44]. Another example is glutamine, which can activate the mTORC1 pathway by being exchanged for leucine via the large neutral amino acid transporter (LAT1) or by producing more α-KG, both of which lead to cell growth and autophagy suppression [21,45,46]. In malignancies reliant on glutamine, synthetic lethal activity can be triggered by inhibiting glutaminolysis. This can be done in conjunction with chemotherapy drugs or other metabolic inhibitors.

### 3.3. Determinants of Glutamine Dependence in Cancer

Several factors were identified to promote glutamine reliance in cancer. For example, some of the most frequent oncogenes in human cancers were found to induce the reprogramming of glutamine metabolism and potentially sensitize tumors to anti-glutaminolysis therapies. This includes aberrations in *c-MYC* [47,48,49], *JUN* [50], *TP53* [41,43], *KRAS* [23,51,52], *RB1* [53], *STK11* [51], *KEAP1* [51,54], *IDH1* [55,56], the PI3K/mTOR axis [34], and FLT3 tyrosine kinase signaling [57]. Moreover, the involvement of glutamine and GLS in redox homeostasis also makes glutamine a crucial player in the cellular handling of oxidative stress. Consistent with this concept, pan-cancer metabolic analysis revealed significant co-dependency on GLS and glutamylcysteine synthetase, suggesting that tumors may rely on glutamine catabolism for its contribution to glutathione biosynthesis [58]. In addition to oncogenetic changes and the need to overcome oxidative stress, the glutamine dependence of cancer could be influenced by the nutrient availability in the tumor microenvironment. Previous studies have demonstrated that environmental cysteine is able to induce a differential dependence of cancer cells on glutamine. A high level of cysteine increases glutamine catabolism and drives glutamine dependence through the action of the cystine/glutamate antiporter xCT [59].

Although glutamine’s role as a carbon donor is crucial in the TCA cycle, an interesting argument states that “true glutamine addiction” only arises when substantial glutaminolysis is required to supply GSH biosynthesis for immediate handling of oxidative stress. This is based on the abundant inter-related pathways that offer endogenous or exogenous supplies of TCA metabolites that could compensate for the anaplerotic role of glutamine [60]. Supporting this perspective, given that pyruvate sustains the TCA cycle via conversion into OAA, cancers that are present in environments with higher levels of pyruvate, such as the lung, show lower dependence on glutamine metabolism. Furthermore, metastatic tumors to the lung also showed diminished glutamine dependence in comparison to their parental cancers [61].

## 4. Development of Anti-Glutaminolysis Drugs

Glutaminolysis has emerged in recent years as an effective therapeutic focus in the treatment of cancer. In order to restrict the proliferation of glutamine-addicted cancer cells, researchers have created various drugs that target different stages in glutamine metabolism.

### 4.1. Glutamine Depletion

Because cancer cells’ reliance on glutamine metabolism is generally greater than in normal tissues, and glutamine deprivation in cancer cell cultures frequently results in cell death, scientists have explored depleting glutamine as a cancer therapy strategy. Multiple compounds, such as bacterial L-glutaminase and phenylbutyrate derivatives, that induce systemic depletion of glutamine were evaluated as potential therapeutic agents [62,63]. However, relevant research has ebbed due to the limitation of these agents, which is the easy acquisition of tumor resistance by de novo glutamine synthesis in cancer or stromal cells. Another strategy to achieve glutamine deprivation in cancer is by depleting sources of glutamine in the tumor microenvironment. Inhibition of glutamine synthesis in cancer-associated fibroblasts was found to limit ovarian tumor growth [64]. Moreover, exosomes derived from the tumor microenvironment were shown to support tumor growth under nutritional stress [65]. However, particular inhibitors of these pathways are not yet available since therapeutically relevant targets have not been unequivocally identified.

### 4.2. Inhibitors of Glutamine Uptake

In one case, the presence of several kinds of glutamine transporters in the plasma membrane made it difficult to accomplish selective blocking of glutamine absorption [66]. Nevertheless, the link between the transporter SLC1A5 and oncogenic MYC expression and adverse prognosis in a variety of cancers prompted the development of the SLC1A5 inhibitor as a target of pharmacological blockage [67,68,69]. L-Glutamyl-p-nitroanilide (GPNA), an analog of glutamine, was developed into a first-generation SLC1A5 inhibitor [70,71]. Despite early studies showing favorable results in limiting tumor growth and producing synergistic effects with other cancer therapies, GPNA has been identified by growing evidence to have low selectivity as a transporter inhibitor [71,72]. Recent research has shown that GPNA can inhibit multiple other amino acid transporters and that GPNA’s inhibition of cell viability is likely due to off-target effects from the activity of the γ-Glutamyltransferase enzyme rather than the disruption of glutamine metabolism [73].

Consequently, V-9302, a small-molecule antagonist derived from GPNA, was subsequently developed. In comparison with GPNA, V-9032 has approximately 100-fold increased potency in blocking cellular glutamine uptake, and inhibition of SLC1A5 with V-9032 was demonstrated to cause cell death, disrupt redox equilibrium, and result in disrupted development and progression of cancer [74]. However, V-9032 faced similar challenges as GPNA in selectivity and off-target effect. The mechanism underlying V-9032′s effect was questioned since the knockout of SLC1A5 did not result in decreased sensitivity to V-9302 [75]. The tumor suppression effect of V-9032 was likely due to the combined blockage of other glutamine transporters, such as SNAT2 and LAT1 [75]. In this regard, a specific SLC1A5 inhibitor remains unidentified. Further investigation is warranted to unveil the mechanism of action and optimize the structure of these lead compounds.

### 4.3. Antagonists of Glutamine

For decades, researchers have been searching for ways to develop effective glutamine antagonists that block glutamine metabolism in cancer cells [76,77,78,79,80]. Compounds such as DON (6-Diazo-5-oxo-L-norleucine), acivicin, and azaserine have been shown to inhibit the growth of a variety of cancers in various clinical studies [17]. However, most of the above glutamine analogs exhibit severe toxicity and are not recognized as favorable cancer medications despite their strong effectiveness in halting the proliferation of cancer cells [12].

In this context, scientists sought to develop DON prodrugs that maximized the transport of DON to tumors while limiting its exposure to gastrointestinal (GI) tissues. Some also proposed administering these prodrugs at a low dose so as to reduce the toxicities associated with the GI system [77,81]. Noticeably, JHU083, a prodrug that releases DON, has received much attention as a novel glutamine antagonist. JHU083 is cleaved by cathepsins and other enzymes in tumors, which helps to reduce the adverse effects that it has on other organs [82]. In addition, treatment of MC38 colon cancer cells with JHU083 not only resulted in a reduction in glycolysis and oxidative phosphorylation but also enhanced the oxidative metabolism in effector T cells and thereby boosted anti-tumor immunity [83]. Taken together, prodrugs of DON, such as JHU083, have been refined in terms of their delivery strategies and doses and may show encouraging results in patients with glutamine-dependent cancers in future studies.

### 4.4. Glutaminase Inhibitors

Glutaminase is an amidohydrolase that catalyzes the conversion of glutamine (GLN) into glutamate (GLU) and ammonium ions in the first step of glutaminolysis. GLS1 is a critical enzyme in the growth and proliferation of many types of cancer and is thus a potential therapeutic target. Two lead compounds, bis-2-(5-phenylacetamido-1,3,4-thiadiazol-2-yl) ethyl sulfide (BPTES) and compound 968, were first developed as allosteric glutaminase inhibitors around a decade ago [84,85]. BPTES is a selective allosteric inhibitor of GLS1. Despite showing high anti-proliferative efficacy in vitro, the application of BPTES in vivo was restricted by its low solubility [86,87]. To address this problem, various delivery strategies and structural modifications were sought to improve its bioavailability [88,89,90]. One of the derivatives, Telaglenastat (CB-839), was found to be a potent hit and has advanced to clinical trials as monotherapy or combination therapy with other treatments [84]. On the other hand, compound 968 is a pan-glutaminase inhibitor with a four-fold higher potency in inhibiting GLS2 than GLS1 [91]. Recently, many other glutaminase inhibitors, such as C9.22 [92], compound 27 (IPN60090) [93], alkyl benzoquinones [94], and thiazolidine-2,4-dione compounds [95] were developed and are undergoing evaluation.

#### 4.4.1. BPTES

The inhibitory effect of BPTES on GLS1 was achieved by affecting the dimer–tetramer equilibrium of GLS1 [96,97]. However, the fact that BPTES has poor solubility (0.144 μg/mL) makes it challenging to administer in vivo [12,87]. Accordingly, novel techniques have allowed the encapsulation of BPTES in sub-100 nm nanoparticles, which demonstrated better pharmacokinetic properties and efficacy compared to unencapsulated BPTES [90]. Further metabolomic analyses revealed that tumor cells that survived after glutaminase inhibition depend on glycolysis and glycogen synthesis for energy production. Consistent with this finding, combination therapy of BPTES nanoparticles and metformin, a mitochondrial complex I inhibitor that blocks glycogen synthesis, resulted in greatly increased inhibition of tumor growth compared to monotherapy with either agent alone [90]. Safety evaluation using a patient-derived pancreatic cancer mouse model found no weight loss or signs of liver or kidney toxicity with the combination of BPTES-NPs and metformin [90]. The combined targeting of multiple metabolic pathways with small-molecule drugs showed remarkable efficacy in disturbing the proliferation of cancer cells in preclinical models and holds promise for future cancer therapy.

#### 4.4.2. CB-839

Although derived from BPTES, the potency and kinetic behavior of CB-839 differed from those of BPTES. The effect of CB-839 is time-dependent and slowly reversible. Compared to BPTES, CB-839 has increased potency and distinct kinetic behavior, showing a slow-on/slow-off mechanism [98]. The early preclinical results of CB-839 were promising. For example, one recent study found that CB-839 had a selective inhibitory effect on PIK3CA-mutant, but not WT, colorectal malignancies [99]. In addition, an additive impact on apoptosis was demonstrated when CB-839 was coupled with 5-FU, camptothecin, oxaliplatin, and regorafenib in HCT116 colorectal cells. These findings showed that the addition of CB-839 to current cancer therapy might greatly improve the treatment of patients with PIK3CA-mutant colorectal tumors [99].

As the most studied glutaminase inhibitor with high expectations, dozens of clinical trials are now underway to evaluate CB-839′s effect in vivo. However, early trials showed inconsistent results. While one phase I (NCT02071862) and one phase II (NCT03163667) trial reported enhanced anti-tumor effects for CB-839 when combined with cabozantinib [100] or everolimus [100,101] in metastatic renal cell carcinoma, a recently published phase II randomized controlled trial (NCT03428217) failed to demonstrate improved progression-free survival with CB-839 + cabozantinib compared to placebo + cabozantinib [102]. Despite the controversial results in efficacy, most trials reported tolerable adverse events in response to CB-839 treatment [100,101,102].

#### 4.4.3. Compound 968

Compound 968 is a pan-glutaminase inhibitor that interacts with both kidney-type glutaminase (KGA) and glutaminase C (GAC) isoforms of GLS1 by preventing the combination of inactive monomers of GLS1 into an active tetramer [92,103]. Moreover, compound 968 has an inhibitory effect on GLS2, which was previously identified as being essential in the tumorigenesis of luminal-type breast cancers [104]. Research on breast cancer cell lines discovered that basal- and luminal-type breast cancer, although both rely on glutamine metabolism, adopt distinct pathways for glutaminolysis [104]. The glutamine utilization in luminal-subtype breast cancers is mediated by GLS2, rendering them insensitive to many commonly used GLS1 inhibitors. In line with this finding, targeting GLS2 with compound 968 successfully suppressed the proliferation and tumorigenesis of BPTES-resistant luminal-type breast cancer [104]. Aside from breast cancers, compound 968 was found to be effective against various cancers in preclinical studies, including endometrial [105], ovarian [106,107], hepatocellular [108,109], non-small cell lung cancer [110], and multiple myeloma [111].

#### 4.4.4. C9.22

Recently, a high-throughput screening study using the coupled enzyme-based fluorescent glutaminase activity assay to screen a library of about 30,000 compounds was conducted [92]. As a result, 11 glutaminase inhibitors were found to be hits, and they were further characterized by in silico, biochemical, and glutaminase-based cellular assays [92]. The structure-activity relationship research on the most promising hit (C9) led to the identification of C9.22, a derivative with improved in vitro and cellular glutaminase-inhibiting activity [92]. C9.22 inhibited GAC selectively, presumably through a mechanism similar to BPTES and CB-839. The new glutaminase inhibitor C9.22, which has a unique structure and prevents cells from using glutamine, leads to suppression of propagation in three dimensions [92].

#### 4.4.5. Combination Therapies

Due to the pivotal role of GLS in glutaminolysis and its influence on multiple inter-related pathways, GLS inhibitors have the potential to exhibit synergistic effects with many cancer therapies that target different pathways. Various combination regimens were proposed. Metformin, an antidiabetic drug that was found to have an anticancer effect in multiple malignancies [112], has a tendency to be more effective against cancer cells that have survived GLS inhibition [113]. Moreover, as inactivation of GLS produces a redox imbalance, reduces the production of nucleotides, and generates replication stress, cancer cells become more dependent on poly (ADP-ribose) polymerase (PARP) DNA repair. This therefore sensitizes them to PARP inhibitors, suggesting the consideration of the combined therapy of GLS inhibitors and PARP inhibitors as a novel therapeutic regimen [19].

On the other hand, it was shown that pancreatic cancers might circumvent GLS inhibition by increasing their glutamate synthesis through the glutaminase 2 route [114]. Inhibition of glutamine transaminase K, a major enzyme of the glutaminase 2 pathway, in conjunction with GLS inhibition, can dampen both metabolic pathways [114]. Another study reported that N-acetylaspartylglutamate can act as a crucial reservoir, supplying glutamate to cancer cells via carboxypeptidase II (GCPII), even when glutamate production from exogenous glutamine is restricted. This makes GCPII a feasible target for cancer therapy, either alone or in combination with GLS inhibition [115]. Taken together, the use of GLS inhibitors in conjunction with other therapies may provide a viable strategy for the effective treatment of cancer.

In recent years, there has been a growing focus placed on the relationship between the inhibition of glutaminase and the activation of the immune system brought about by immunotherapies. As a glutamine-rich tumor microenvironment was suggested to be essential for CD8 T cell activation and effector function, the combination of glutamine inhibition with immunotherapy was called into question [116,117]. This was corroborated by a recent study, which demonstrated that glutaminase inhibition with CB-839 impaired the clonal expansion and activation of CD8 T cells in *Lkb1*-deficient lung cancer [116]. Although glutaminase inhibition or anti-PD1 immunotherapy may be beneficial to this subset of cancer as monotherapy, their combination could lead to a contradictory effect [116]. Since several clinical trials have been launched to examine the combination of CB-839 with anti-PD1 immunotherapies (NCT03894540, NCT04265534, and NCT02771626), the importance of potential interference should be carefully considered.

## 5. Glutaminolysis in CSCs

CSC metabolic features have been the subject of intense research with the goal of elucidating the mechanisms underlying their aggressive behavior and stemness traits. Upregulated features of glutaminolysis have been demonstrated in multiple lines of CSCs, including pancreatic, lung, ovarian, cervical, prostate, and hepatocellular cancers, suggesting their reliance on glutamine metabolism and potential sensitivity to anti-glutaminolysis therapies [118,119,120,121,122].

### 5.1. Glutamine Dependence in CSCs

Research demonstrated lower uptake of glucose in ovarian clear cell carcinoma with CSC-like properties on fluorodeoxyglucose positron emission tomography/computed tomography, indicating a low activation of glycolysis [123]. Further in vitro evaluation found that, in comparison to non-CSCs, CSCs have lower expression levels of LAT1, a transporter of glutamine efflux, and GLUT1, a transporter of glucose influx [123]. The expression level of SLC1A5 shows no difference, suggesting a comparable glutamine influx between CSCs and non-CSCs [123]. Taken together, these lines of evidence depict a glutamine-accumulating feature and indicate the metabolic diversion toward glutaminolysis from glycolysis in ovarian CSCs (Figure 2). Additionally, a magnetic resonance imaging (MRI) assessment demonstrated that there is a strong positive correlation between the absorption of glutamine and the expression of CSC markers [124]. For instance, CD44 contributes to redox control by substantially activating xCT and stabilizing the subunit of xCT (Figure 2), which functions as an antiporter that mediates the diversion of gulatmine metabolism toward glutathione biosynthesis [124]. This suggests that the association between glutamine uptake and CD44 may be connected to the function of xCT [124]. Other research also pointed out that glutamine addiction might be explained by the functional coupling of xCT and SLC1A5 [125]. The proposed theme is that when glutamine is taken up by cancer cells via SLC1A5, it is converted into glutamate by GLS [124]. After that, the xCT system transfers glutamate out of the cell in exchange for cystine in order to keep the amount of glutathione sustainable [124]. This highlights that the dependence of CSCs on glutamine may not necessarily stem from the participation of glutamine in the TCA cycle and energy metabolism but also from its pivotal role in the maintenance of redox balance.

### 5.2. Oncogenotypes That Drive Glutamine Dependence, Stemness, and Treatment Resistance in CSCs

The glutaminolysis of CSCs has been inextricably linked to certain metabolic regulators, including the oncogenic alterations of *MYC*, *KRAS*, and *TAp73* [126,127,128]. This has the important implication that the metabolic reprogramming toward glutaminolysis in CSCs is not simply a passive adaptation to meet proliferative needs, instead, it is a complex phenomenon that is also driven by the intrinsic genetic factors that orchestrate the oncogenesis. The resulting metabolic alteration is a hallmark of cancer and has been recognized as a prerequisite step for the induction of the CSC phenotype [129].

#### 5.2.1. c-MYC

Different genomic aberrations were found to drive glutaminolysis via distinct mechanisms. In the case of *c-MYC*, glutamine absorption was increased by overexpression of glutamine transporter SLC1A5, and subsequent catabolism of glutamine was enhanced via stimulation of the transcription of glutamine synthetase and GLS [29,37]. Consistently, knockdowns of SLC1A5, GLS, and c-MYC were demonstrated to constrain CSC characteristics [113]. Moreover, overexpression of *MYC* has been associated with radioresistance in CSCs via GLS-mediated glutamine catabolism [122]. Interesting research corroborated this concept by demonstrating that combined glutamine deprivation and irradiation led to a selective radioresistance of *MYCN*-amplified neuroblastoma cells [130]. Further experiments indicate that this phenomenon is manifested through the enhanced capability of *MYCN*-amplified cells to maintain GSH redox homeostasis, promote stemness properties, and regulate DNA repair pathways [130]. In contrast, glutamine deprivation increased radiation sensitivity and decreased CSC properties in non-*MYCN* amplified cells [130]. Similar results were observed in prostate cancer. Radioresistant prostate cancer and prostate CSCs were found to have high glutamine requirements [122]. In these cells, deprivation of glutamine or inhibition of *MYC* or GLS led to resensitization to radiotherapy, while the same regimen did not pose a toxic effect on nonmalignant prostate cells [122]. Supporting these findings, upregulation of MYC and GLS was associated with a significantly reduced progression-free survival in prostate cancer patients undergoing radiotherapy, which could be attributed to an increased population of CSCs and radioresistant cells [122]. The same study also revealed that the activation of ATG5-mediated autophagy, which is a GLS-driven cellular response to glutamine deficiency, is an important survival strategy that endows the tumor with the ability to overcome radiation-mediated cell damage [122]. This supports the use of anti-glutaminolysis drugs in conjunction with autophagy blockage as a potential regimen for radiosensitization of prostate cancer and elimination of prostate CSCs, with promising results validated in a subsequent investigation [13].

#### 5.2.2. KRAS

Another oncogenic change that drives stemness and chemoresistance is *KRAS*, one of the most prominent genetic aberrations in gastrointestinal cancers [131]. A mutation of *KRAS* promotes glutaminolysis through the actions of glutaminase and SLC25A22, a mitochondrial glutamate carrier that transfers glutamate into the mitochondria to participate in the TCA cycle [132]. This resulted in succinate accumulation and the subsequent initiation of a cascade of epigenetic alterations, including increased DNA methylation, enhanced Wnt/β-catenin signaling, and LGR5 expression [132]. These changes ultimately resulted in the proliferation of tumor cells with increased stemness and resistance to the chemotherapy agent 5-FU [132]. Subsequent in vitro and in vivo studies demonstrated that the knockdown of SLC25A22 reversed the sequential effect of *KRAS* on the TCA cycle and resensitized colorectal cancer cells to chemotherapy [132]. In pancreatic ductal adenocarcinoma (PDAC), another *KRAS*-mediated signaling pathway was proposed to support glutamine metabolism and the development of chemoresistance [126]. Mutant *KRAS* was found to promote glutaminolysis via upregulation of the transcription factor NRF [126]. NRF is a major stimulator of GSH synthesis that promotes glutamine use by increasing the expression of multiple relevant enzymes, such as ME1 and gamma-glutamyl-cysteinyl-ligase, a rate-limiting enzyme of GSH synthesis [133]. This in turn contributed to enhanced antioxidation capacity and chemoresistance in *KRAS*-mutant PDACs. Conversely, disruption of glutamine metabolism sensitizes *KRAS*-mutant PDACs to gemcitabine, suggesting a potential for exploiting anti-glutaminolysis treatments to improve chemotherapy efficacy [126].

#### 5.2.3. TAp73

The tumor suppressor TAp73, a structural homologue of p53, has been studied intensely for its role in cancer metabolic regulation and cell proliferation [134,135]. Recently, TAp73 was found to be essential for the maintenance of stemness and self-renewal capacity in CSCs [128]. In the context of embryonal carcinoma stem-like cells and breast CSCs, knockdown of TAp73 profoundly diminished the expression of stemness genes and tumorigenic capacity both in vivo and in xenografted mice [128]. Mechanistically, this is manifested via the regulation of the glutamine transporter SLC1A5, GLS, and the proline-glutamine-ornithine axis, which has an impact on the redox capacity of CSCs. Deficiency of TAp73 led to the aggravation of glutamine depletion, the accumulation of reactive oxygen species, and the differentiation of CSCs into normal cancer cells. This was demonstrated by a significantly lowered expression of differentiation marker β3-tubulin and pluripotency factors Oct4, Nanog, and Sox2 [128]. Collectively, this suggested a clinically relevant difference between differentiated cancer cells and CSCs, in which TAp73-mediated glutamine metabolism could serve as a therapeutic target for the elimination of CSCs.

### 5.3. GLS1 in CSCs

Despite the fact that there has not been a universal consensus on the metabolic hallmark of CSCs, it has been increasingly accepted that the acquisition and maintenance of stemness are metabolism-dependent processes [136]. Accumulating evidence suggests that for the CSCs to preserve stemness qualities, it is critical to keep ROS at a low level [136,137,138]. Given the essentiality of ROS handling in CSCs, GLS1, a major regulator of redox homeostasis, has been proposed to attenuate stemness qualities [121]. In line with this concept, high GLS1 expression has been associated with poor prognosis in many, but not all, cancers, including breast, esophageal, liver, head and neck, and blood cancers [121,139,140]. Moreover, glutamine has been reported to play a prominent role in maintaining the stemness of lung and pancreatic CSCs through the regulation of intracellular ROS levels [141]. GLS1 inhibition was shown to suppress the expression of stemness markers in vitro and tumorigenesis capacity in vivo in head and neck squamous carcinoma [142]. A recent study in hepatocellular carcinoma (HCC) found that GLS1 upregulation is associated with stemness and aggressive clinical and histological features [121]. According to additional research, GLS1 regulates stemness in HCC via ROS/Wnt/β-catenin signaling [121]. Inhibiting GLS was shown to suppress β-catenin localization to the nucleus and reduce stemness gene expression in HCC CSCs. Furthermore, the author demonstrated that increased intracellular ROS resulted in decreased β-catenin expression. These findings point to the existence of a positive feedback relationship between GLS and Wnt/β-catenin signaling, in which GLS expression is stimulated by the Wnt pathway and the reduced level of ROS following GLS activation allows β-catenin upregulation and localization [121]. This finding added to previous evidence that Wnt/β-catenin signaling is involved in redox homeostasis via glutamine metabolism regulation in lung and liver cancers [141,143,144]. Taken together, the above evidence provided a good insight into the mechanism underlying the metabolic phenotype of CSCs and indicated the feasibility of targeting GLS1 as a novel strategy to eliminate CSCs.

## 6. Glutaminolysis-Specific Intervention for CSCs

As CSCs differ from parental cancer cells in their glutamine utilization, the first question that arises is: do cancers and CSCs respond differently to anti-glutaminolysis drugs? Research has displayed that the heterogeneity in intrinsic properties could lead to distinct responses to glutaminolysis inhibition among different subtypes of breast cancer and CSCs [145]. In a luminal breast cancer cell line, DON significantly increased cellular apoptosis and decreased cell viability, whereas basal breast cancer cells responded by undergoing a drastic morphological change into an epithelial-like appearance. On the other hand, both luminal and basal breast CSCs demonstrated impaired tumor sphere formation, decreased stemness gene expression, and differentiation into non-CSCs in response to DON treatment, while basal breast CSCs further displayed a profound phenotype change similar to those seen in parental cancer cells, accompanied by a downregulation of mesenchymal markers and an upregulation of epithelial markers [145]. This implies that despite the fact that parental cancer cells and CSCs may have different metabolic features and rely on glutamine for distinct purposes, glutaminolysis pathways may be essential for both populations. In contrast to many chemo- and radiotherapy-based strategies that could be evaded by CSCs [146,147], therapeutic targeting of glutamine metabolism, which is closely linked to the regulation of stemness properties, has the potential to decrease the CSC population in a cancer scenario.

Given the critical role of glutamine metabolism in CSCs, various research has examined the sensitivity of CSCs to glutaminolysis-targeting intervention. In head and neck squamous cell carcinoma, inhibition of GLS activity with the glutamine antagonist DON resulted in decreased tumor sphere formation and abrogation of stemness properties [142]. Meanwhile, as GLS1 is postulated to be a key regulator of the fidelity of CSCs, GLS inhibitors are the most selected drugs for CSC treatment. Compound 968 and CB-839 were able to successfully dampen the in vitro clonogenicity of glioblastoma stem-like cells (GSCs) in neurosphere colonies, resulting in a significant reduction in the number of GSCs [148]. Further investigation of the mechanism was performed by another study, which showed that the sensitivity to CB-839 in GSCs was not due to the contribution of glutaminolysis to the TCA cycle; rather, the sensitivity was brought on by reduced intracellular glutamate, which led to the amino acid deprivation response (AADR) [149]. Continuing this line of research, a modification of the CB-839 delivery route was developed by loading the drug into PEGylated gold nanoparticles with conjugated CD133 aptamers (Au-PEG-CD133-CB-839) [150]. The conjugation of CD133, a surface marker recognized on CSCs, was intended to improve the targeting of this drug to CSCs. Making use of the small size and high biocompatibility of gold nanoparticles, this design contributes to improved permeability of the BBB and penetration of the tumor. A preliminary in vitro study demonstrated that Au-PEG-CD133-CB-839 significantly decreased the viability of CD133-positive cancer cells in comparison with treatment with CB-839, in which a dose-dependent effect was seen [150]. This highlighted a feasible strategy for the targeting of CSCs, while further studies in cells that are not restricted to CD133-positive populations are warranted to validate its effect.

In addition, CB-839 and compound 968 were tested on TNBC CSC cell lines. A 70–80% reduction of stemness markers was seen on flow cytometry following 3 days of treatment. Moreover, CB-839 elicited a dose-dependent inhibition of cell viability by over 80% in the two tested cell lines. The tumor sphere formation ability was also significantly reduced by CB-839 or compound 968, suggesting a potential utility of GLS inhibitors in TNBC [151]. The combination of metformin and compound 968 was also found to result in an increased effect of stemness suppression in colorectal CSCs. This combination demonstrated similar effects in xenografts as well as those seen in vitro and in human cancer organoids [113].

Taken together, while most studies are still preclinical, accumulating evidence has verified the possibility of harnessing GLS inhibitors in the eradication of CSCs and combating cancer with treatment resistance.

## 7. Future Perspectives

In CSCs with a high degree of metabolic flexibility, it is still necessary to draw a full picture of the peculiarities of glutaminolysis. We envisage that glutanimolysis-based therapy would ultimately be adopted as an add-on to traditional anticancer therapy, especially for the prevention of tumor recurrence and metastasis. Thoroughly examining the role of glutaminolysis in CSCs can give hints for fighting CSCs through metabolic intervention. Furthermore, the difference in glutaminolysis that exists between CSCs and regular stem cells has to be identified so that patients can be cured without their normal tissues being damaged. As a result, the glutamine metabolism of CSCs needs to be explored in more detail, and the mechanism of metabolic adaptation has to be uncovered. Once the metabolic pathways of CSCs have been clearly elucidated, novel metabolic therapeutic interventions will be able to be developed to couple with existing cytotoxic regimens. This will allow for the more effective elimination of CSCs, the prevention of recurrence and metastasis of cancer, and the eventual cure of cancer patients.

## 8. Conclusions

CSCs are dependent on glucose, glutamine, and a variety of other substrates to a greater extent than their non-cancerous counterparts. GLS expression in CSCs can determine their response to GLS-inhibiting drugs. Therefore, biomarkers such as GLS expression level can be used to select patients who are most likely to respond to and benefit from anti-GLS therapy [152]. Given that glutamine metabolism is at the root of human cancers and that GLS expression in cancer cells can discern their response to GLS-inhibiting drugs, an increasing number of studies have investigated the metabolic pathways in CSCs and the possibility of using them as a new target for anticancer drugs. However, the metabolic plasticity of CSCs hinders the effectiveness of current metabolism-targeting drugs. CSCs are cells that have the potential to differentiate into other cell types. As a result, there is a need for research and development of medications that target numerous metabolic pathways at the same time. In the future, researchers will examine the molecular and metabolic vulnerabilities of cancer cells. This will make it possible to stratify additional patient populations that are resistant to cancer treatment but could benefit from GLS inhibitor treatment in combination with other anti-cancer drugs. Combination therapy is by far the most successful form of cancer treatment [153], due to the fact that it inhibits multiple separate pathways at once, reducing the possibility of the creation of cancer cells that are resistant to treatment. Innovative cancer treatment methods will be further highlighted, and their potential will be further potentiated as a result of future research that will reveal more mechanistic insights into the metabolic adaptation of glutamine-dependent malignancies.

## Data Availability

Not applicable.
